# Amniotic Membrane Versus Platelet-Rich Fibrin for the Treatment of Gingival Recession Using the Vestibular Incision Subperiosteal Tunnel Access Technique: A Randomized Controlled Clinical Trial

**DOI:** 10.7759/cureus.103991

**Published:** 2026-02-20

**Authors:** Hoshang Advani, Ashutosh Dixit, Shambhavi Kumar, Kamal Pandey, Seema Dixit, Subash Mehta, Nancy Neha, Dany B Philip

**Affiliations:** 1 Dentistry, All India Institute of Medical Sciences, Rishikesh, Rishikesh, IND; 2 Conservative Dentistry and Endodontics, Seema Dental College and Hospital, Rishikesh, Dehradun, IND

**Keywords:** amnion membrane, minimally invasive, prf, recession, root coverage, tunnelling

## Abstract

Background

Gingival recession (GR) results in significant functional and cosmetic concerns and manifests as a disintegration of soft and hard tissues. Recently, clinical research has increasingly focused on the development of minimally invasive surgical approaches that optimize clinical outcomes, alongside the incorporation of biomaterials capable of providing appropriate biological cues to support regenerative potential. Our study aimed to compare the effectiveness of the vestibular incision subperiosteal tunnel access (VISTA) technique, either with amniotic membrane (AM) or platelet-rich fibrin (PRF), for the treatment of Cairo’s RT 1 GR.

Methods

Seven systemically and periodontally healthy patients with Cairo's RT-1 GR at more than two adjacent teeth were recruited for the study. Patients were randomly assigned to Group I (PRF) and II (AM) (31 recession sites each). All recessions were treated with VISTA. Clinical parameters were measured at baseline and one, three, and six months after surgery, including pocket probing depth (PPD), recession height (RH), recession width (RW), width of keratinized gingiva (WKG), relative attachment level (RAL), and mean root coverage percent (MRC).

Results

All clinical parameters demonstrated statistically significant differences in both groups from the pre-operative to the six-month follow-up phase. Except for PPD, there was no statistically significant difference between the two groups. In Group I, there was a significant increase in PPD after one month, which decreased at the six-month follow-up, while in Group II, there was a decrease in PPD from baseline to the six-month follow-up.

Conclusion

Both PRF and AM are effective in managing GR. Although AM showed slightly better results, it was statistically insignificant.

## Introduction

A confident smile is a key indicator of health and self-assurance, but can be compromised by gingival recession, disrupting the harmony between teeth and surrounding tissues, leading to esthetic concerns. Recession is characterized by the apical shift of the gingival margin due to various factors [1], leading to impaired aesthetics, increased sensitivity, root caries, tooth discoloration, and fear of tooth loss [2]. It can impact multiple tooth surfaces and be localized or generalized. Recession occurs due to direct or distinct disruptions of the mucogingival complex, with contributing factors including a thin gingival biotype, tooth malposition, aggressive brushing, smoking, plaque accumulation, and iatrogenic influences.

Periodontal plastic surgery addresses recession with the goals of improving soft tissue quality or achieving root coverage. Techniques like connective tissue grafts, coronally advanced flaps (CAF), and subepithelial connective tissue grafts (SCTG) are used, with CAF combined with SCTG being the gold standard. However, SCTG has limitations such as patient discomfort and limited tissue availability [3,4].

In 2011, Zadeh introduced the vestibular incision subperiosteal tunnel access (VISTA) technique, combined with SCTG, for recession treatment. VISTA minimizes tissue damage, preserves blood circulation, and reduces gingival margin displacement [5]. Wound healing post-surgery involves various cells, including platelets, which are crucial for tissue regeneration [6]. Platelet-rich fibrin (PRF) is a newer, cost-effective option that enhances healing by promoting cellular migration and wound healing [7]. PRF also acts as a biological glue, maintaining surgical flaps in place and offering an alternative to connective tissue grafts [8,9]. A recent systematic review and meta‑analysis reported that injectable platelet‑rich fibrin can modify gingival phenotype and improve soft‑tissue outcomes, reinforcing the biological rationale for using platelet concentrates in mucogingival surgery. Amniotic-based allografts, particularly dehydrated allograft amniotic membrane, have emerged as a promising option in periodontal surgery, offering excellent root coverage, tissue thickness improvement, and natural aesthetic outcomes [10].

To date, there is a lack of clinical studies comparing PRF to amniotic membrane using a minimally invasive technique for treating gingival recessions. This study investigates the impact of growth factors on post-mucogingival surgical wound healing, comparing the effects of PRF and AM with VISTA using a split-mouth model.

The primary objective of the study was to assess the change in aesthetic root coverage, which was done by measuring recession height (RH), recession width (RW), and pocket probing depth (PPD) following treatment of GR by VISTA with PRF or AM. The secondary objectives of the study were to assess the changes in clinical parameters following intervention for gingival recession, including the width of keratinized gingiva (WKG), relative attachment level (RAL), and mean root coverage (MRC). All the measurements were done using a University of North Carolina-15 probe (UNC 15) with customized acrylic stents.

## Materials and methods

Study design and ethical approval

This single-blind, randomized controlled clinical trial included patients aged 18-70 years presenting with Cairo’s RT1 gingival recession defects [11]. Participants were recruited from the Department of Dentistry (Periodontics), All India Institute of Medical Sciences (AIIMS), Rishikesh, a tertiary-level healthcare center, after receiving detailed information regarding the study procedures and providing written informed consent in accordance with the Declaration of Helsinki (1964; amended 2024). Ethical approval was obtained from the Institutional Ethics Committee and Review Board prior to study initiation (No. 292/IEC/PGM/2022). The trial was prospectively registered with the Clinical Trials Registry of India (CTRI/2022/11/047450).

Participants

Systemically healthy adults aged 18-70 years presenting with Cairo’s RT1 gingival recession defects involving at least two teeth were screened for eligibility. Participants demonstrated good oral hygiene, absence of tooth mobility, adequate attached gingiva, and sufficient vestibular depth.

Inclusion criteria

Adult patients aged between 18 and 70 years with good systemic health, presenting with at least two teeth affected by Cairo’s RT1 gingival recession, no tooth mobility, adequate width of attached gingiva, sufficient vestibular depth, and overall periodontal health were included in the study.

Exclusion criteria

Patients presenting with systemic or medical conditions that could affect the periodontium, non-vital teeth or untreated periodontal disease, poor oral hygiene, excessive tooth crowding or malalignment, use of immunosuppressive medications or drugs known to cause gingival enlargement, active infections, bleeding disorders, or a high frenal attachment were excluded from the study; additionally, pregnant or lactating women and individuals with a history of smoking were not included.

Sample size calculation

**Figure 1:**
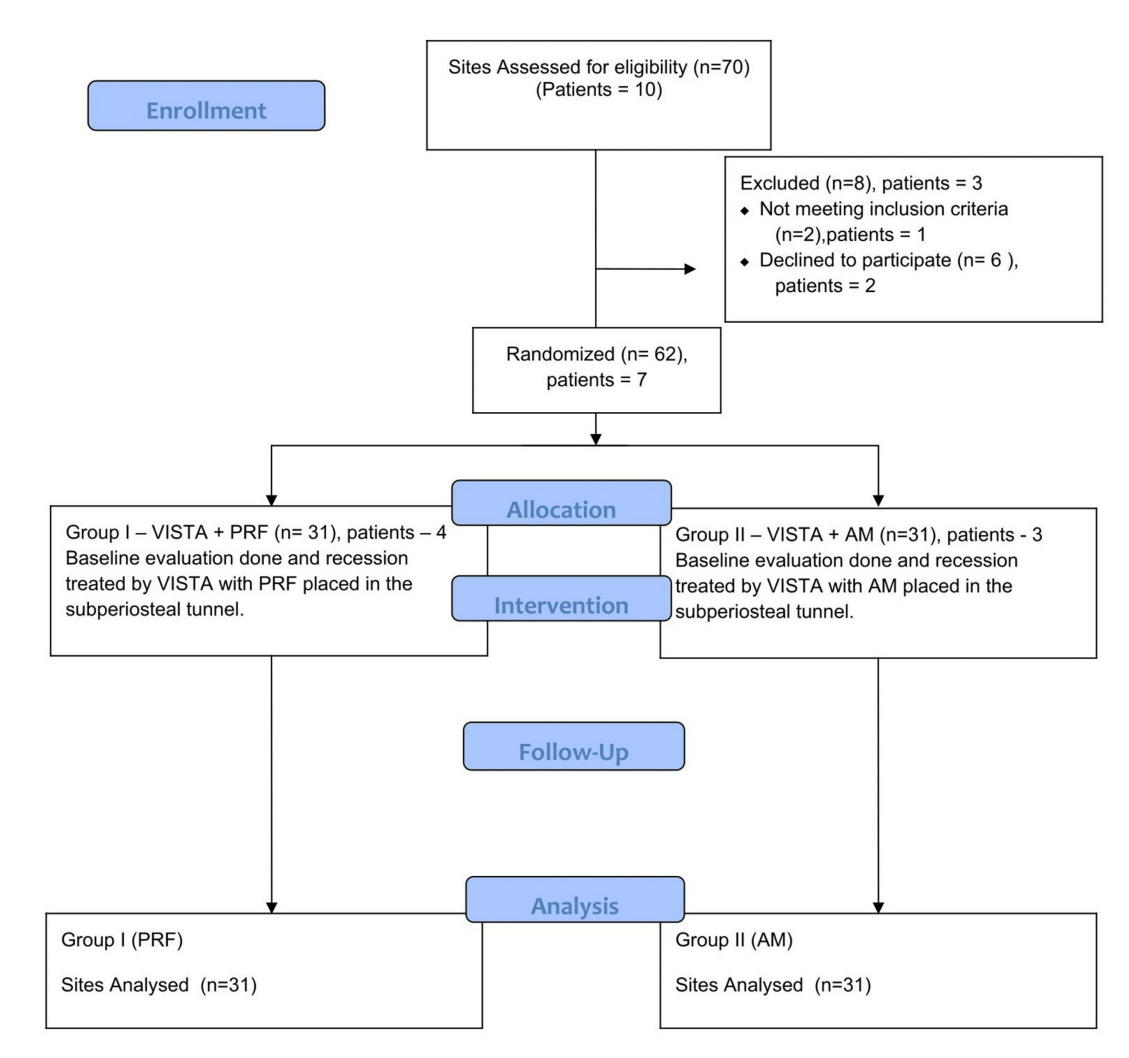
CONSORT flow diagram depicting the methodology, including enrollment, allocation, intervention, follow-up, and analysis of the patients involved in the study CONSORT: Consolidated standards of reporting trials; PRF: Platelet-rich fibrin; AM: Amniotic membrane; VISTA: Vestibular incision subperiosteal tunnel access

A total of 62 gingival recession sites were enrolled and randomly allocated into two parallel groups (31 sites per group) using a computer-generated randomization sequence; an a priori sample‑size calculation based on an expected MRC of 80% with α=0.05 and power=80% yielding N=31 sites. All eligible participants initially underwent Phase I periodontal therapy consisting of scaling and root planing. Only patients demonstrating adequate plaque control and maintaining optimal oral hygiene after one week were included for surgical intervention. In Group I, gingival recession defects were treated using the VISTA technique combined with PRF, whereas Group II received VISTA in conjunction with AM. The study procedures were conducted according to the CONSORT guidelines illustrated in Figure 1.

Randomization and allocation concealment

Eligible recession sites were randomly assigned to either: Group I: VISTA with PRF; Group II: VISTA with AM. Randomization was performed using a computer-generated random allocation sequence prepared by an independent investigator not involved in treatment or outcome assessment. Allocation concealment was ensured using sequentially numbered, opaque, sealed envelopes, which were opened only at the time of surgery.

Blinding

Due to the nature of the surgical interventions, operator blinding was not feasible. However, (i) Participants were blinded to group allocation. (ii) The clinical examiner recording outcomes was blinded to treatment assignment. (iii) Statistical analysis was performed using coded group data to maintain assessor blinding.

Clinical measurements

Clinical measurements were recorded using a University of North Carolina-15 periodontal probe (PCPUNC156, Hu-Friedy, Chicago, IL, USA) by a calibrated, blinded examiner using standardized measurement protocols.

The primary outcome measures included recession height (RH), recession width (RW), and pocket probing depth (PPD). RH was defined as the distance from a fixed reference point on a customized acrylic stent to the crest of the marginal gingiva. RW was measured as the maximum mesiodistal dimension of the recession defect on the labial surface. PPD was recorded as the distance from the gingival margin to the base of the gingival sulcus.

The secondary outcome measures included width of keratinized gingiva (WKG), relative attachment level (RAL), and mean root coverage (MRC). WKG was measured as the distance between the mucogingival junction and the free gingival margin. RAL was defined as the distance from the fixed reference point on the acrylic stent to the most apical extent of the gingival sulcus. Mean root coverage was calculated using the following formula: MRC (%) = [(preoperative recession height − postoperative recession height) / preoperative recession height] × 100.

**Figure 2:**
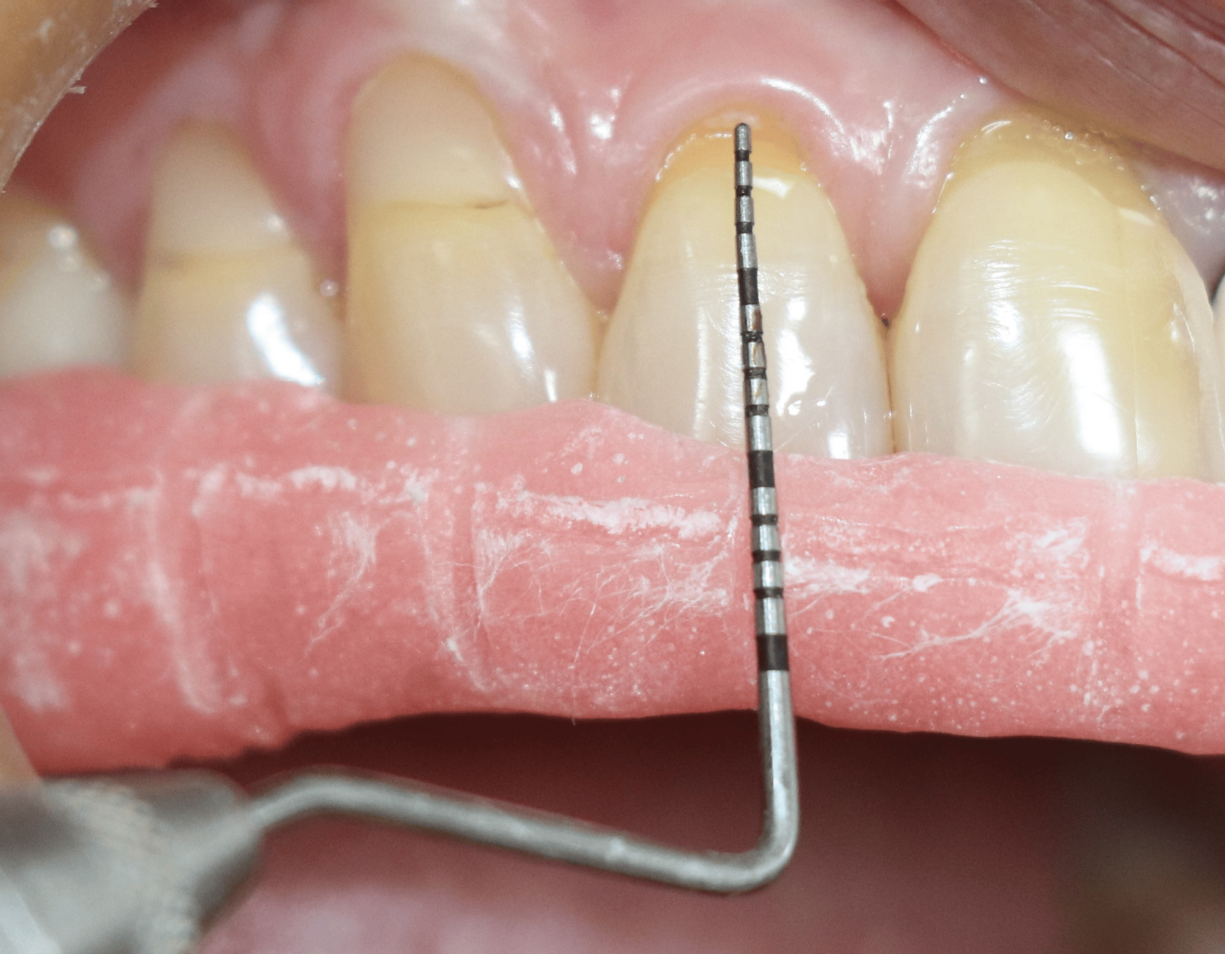
Recession measured with UNC - 15 probe* and occlusal stent with vertical and horizontal grooves used for measurements * - (University of North Carolina-15 periodontal probe Hu-Friedy, Chicago, IL, USA)

All clinical parameters were recorded at baseline and at one, three, and six months postoperatively. To ensure reproducibility and minimize measurement bias, individualized self-cured acrylic stents with an occluso-apical guiding groove corresponding to the mid-buccal aspect of the tooth were fabricated and used as fixed reference guides for probe placement at all evaluation visits (Figure 2).

Surgical procedure

**Figure 3:**
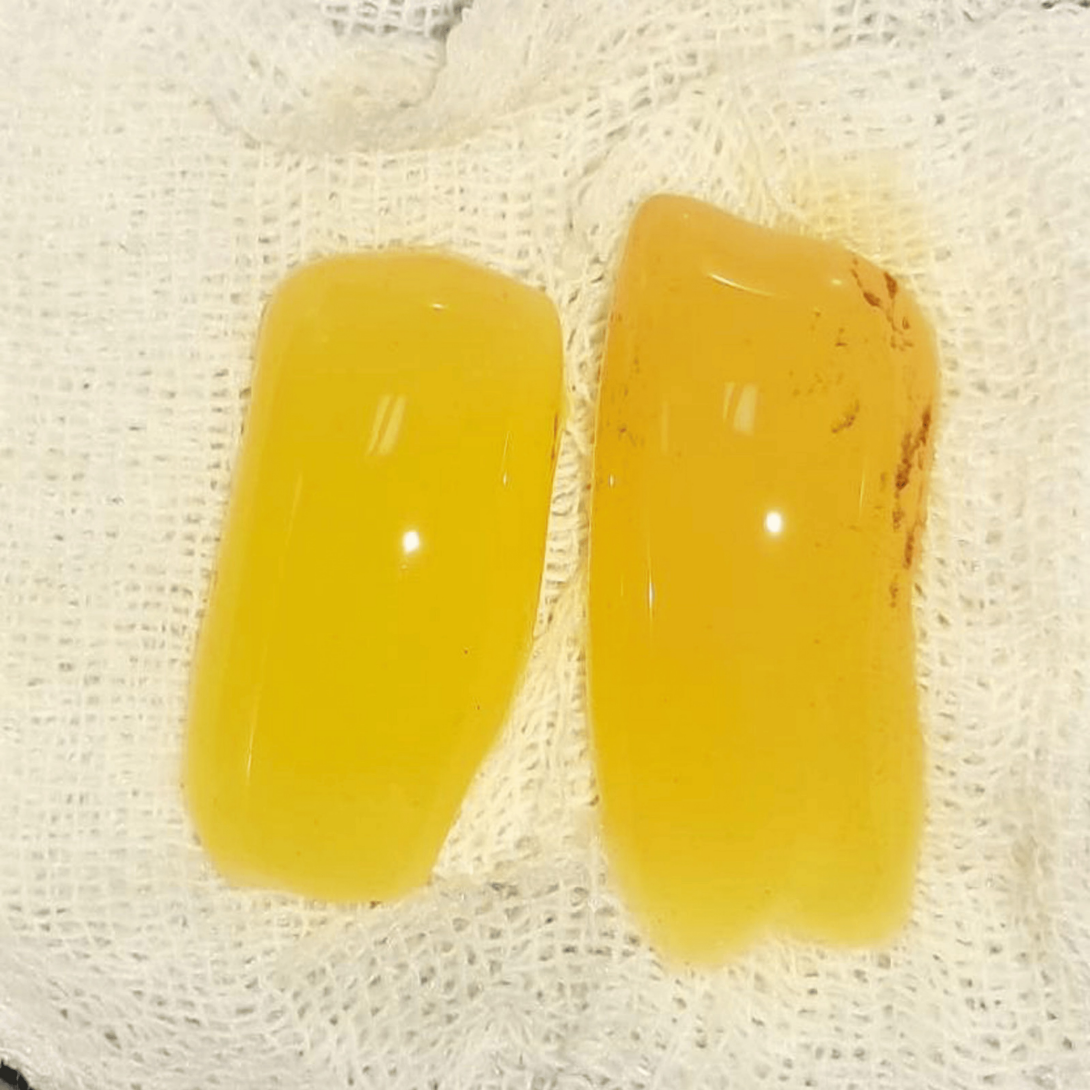
Platelet-rich fibrin (PRF) membrane

**Figure 4:**
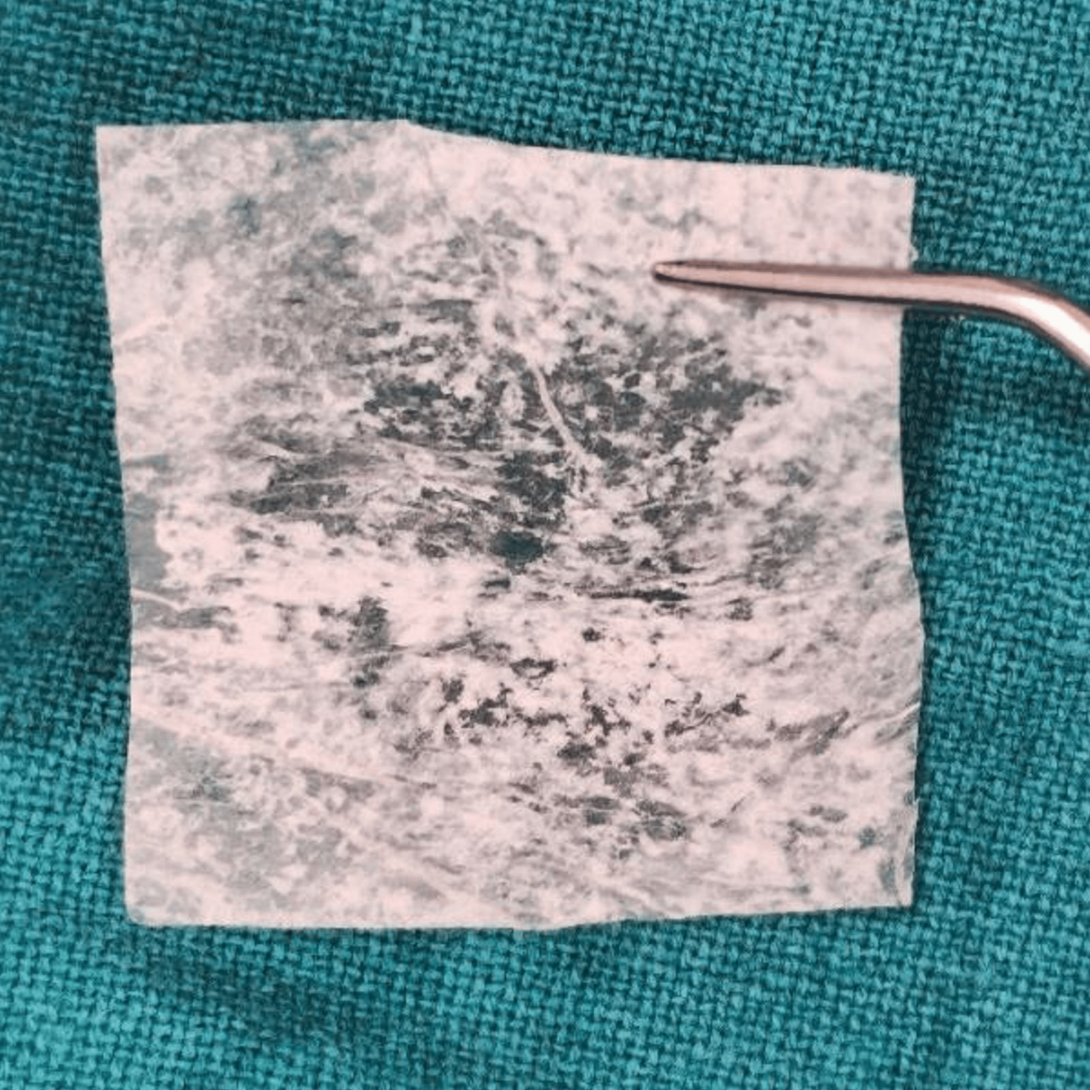
Freeze-dried, irradiated amniotic membrane

**Figure 5:**
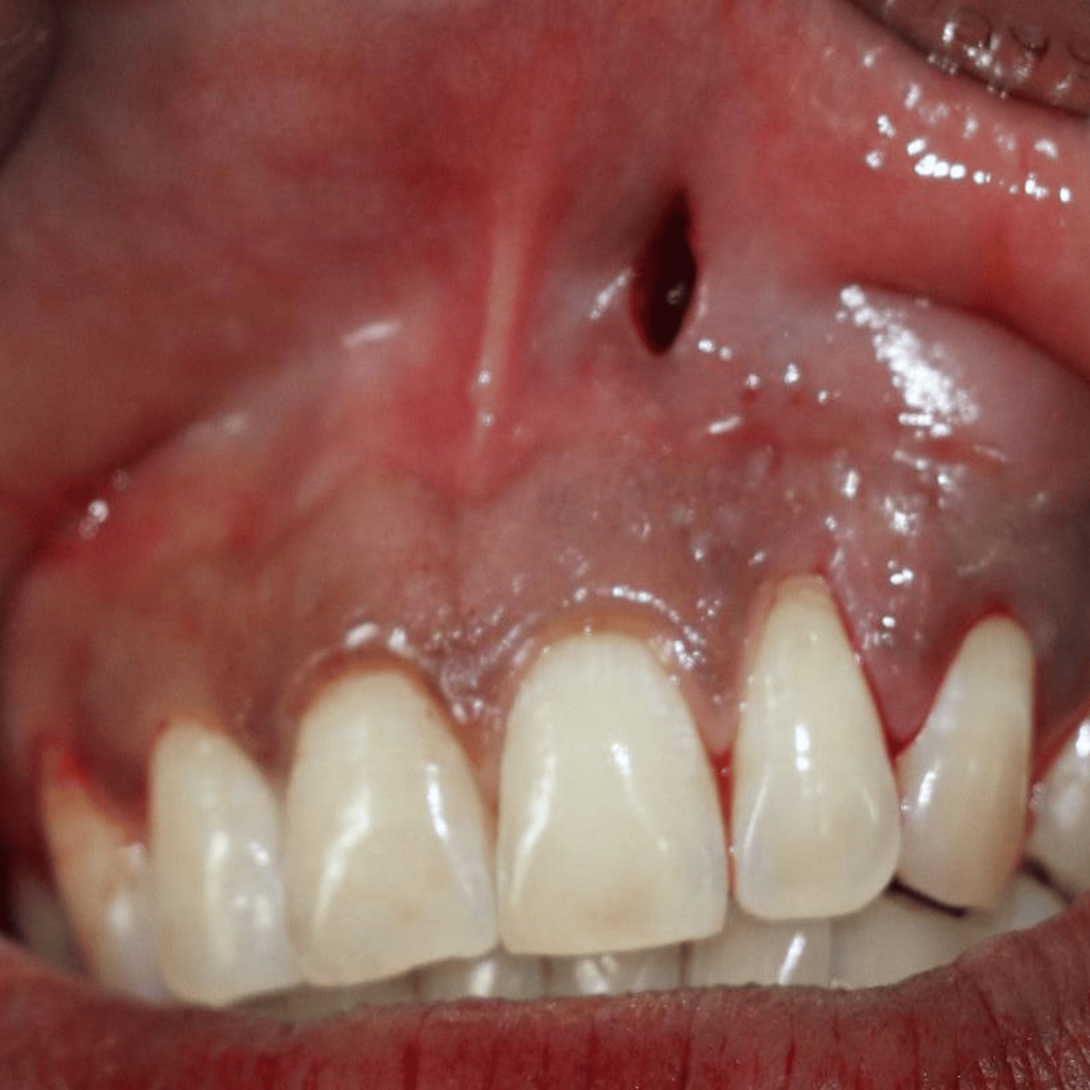
The vertical access incision

All surgical procedures were performed by a single experienced periodontist under local anesthesia using the vestibular incision subperiosteal tunnel access (VISTA) technique. The selected surgical sites were anesthetized using 2% lignocaine hydrochloride containing 1:200000 adrenaline after scrubbing the area with betadine. Selected sites were subjected to surgical intervention by the VISTA technique; in Group I PRF (Figure 3) was placed, and in Group II AM (freeze-dried irradiated membrane, measuring 4*4 cms) (Figure 4) was placed for root coverage. In both groups, the preparation of the recipient site was common using the VISTA technique given by Dr. Zadeh [5]. With the help of a scalpel, a vestibular vertical access incision (8-10 mm long) was made on the mucous membrane extending till the periosteum. The incision began from the mobile mucosa to the apical end of the keratinized gingiva, at least 5 mm from the gingival margin. (Figure 5). The incision was made through the periosteum to elevate a subperiosteal tunnel, exposing the facial osseous plate. This tunnel was extended at least one tooth beyond the tooth requiring the root coverage to mobilize the gingival margins and facilitate coronal repositioning.

Finally, after the dissection was completed, a rolling maneuver was done. This maneuver consisted of inserting the instrument into the subperiosteal tunnel and rolling it towards the gingival margin to stretch the periosteum, allowing tension-free coronal placement of the gingival margin to cover the recession defects. Following this, for Group I, PRF was placed, and AM was placed for Group II.

**Figure 6:**
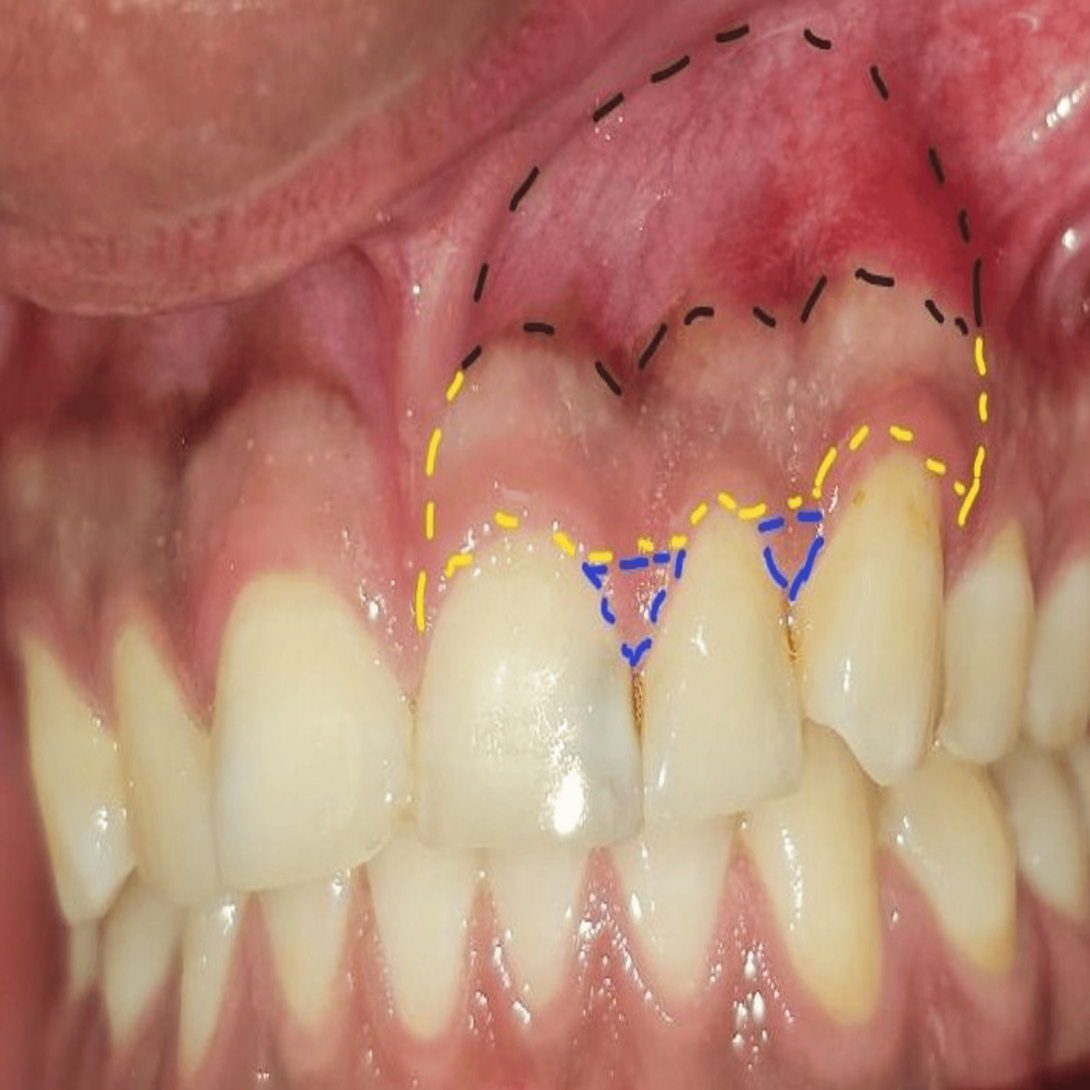
The three zones of the subperiosteal tunnel. A zone: black dotted line; B zone: yellow dotted line; C zone: blue dotted line

**Figure 7:**
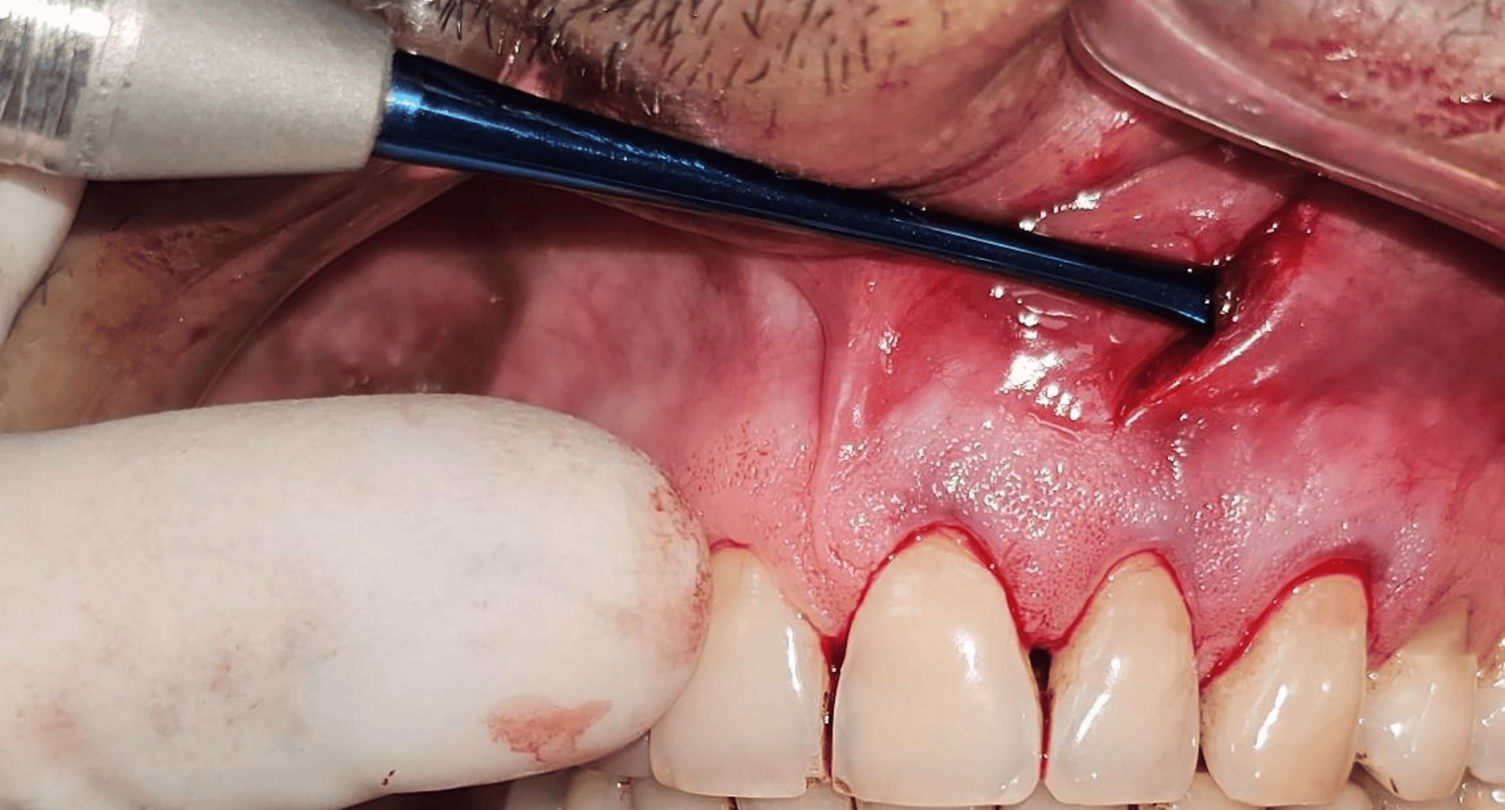
Subperiosteal tunnel dissection in zone A

**Figure 8:**
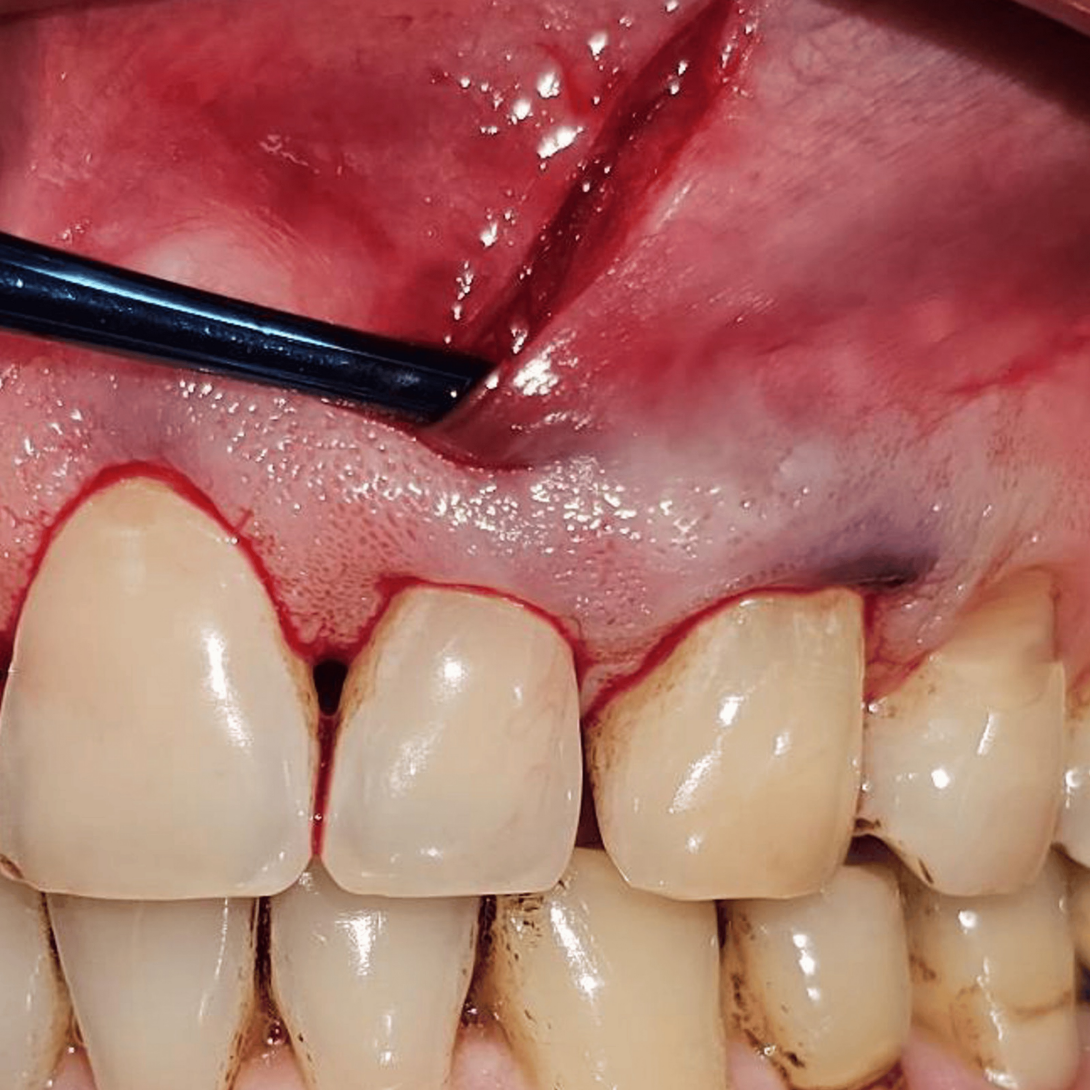
Dissection extending beyond the MGJ into the attached gingiva. Tension-free coronal advancement of the gingival margin can also be appreciated MGJ: mucogingival junction

Briefly, the surgical site extending from the vestibule to the gingival margin is divided into 3 zones: A, B, and C. The A zone extends from the vestibule to the mucogingival junction. The attached gingiva between the mucogingival junction and the gingival margin makes up the B zone, and the papillary complex is known as the C zone (Figure 6). A microsurgical periosteal elevator was first used to create the subperiosteal tunnel in the A zone. The VISTA elevator was introduced through the vestibular access incision and inserted between the periosteum and bone to elevate the periosteum, creating the subperiosteal tunnel (Figure 7). After completing the dissection in the A zone, the tunnel elevation was extended into the B zone beyond the mucogingival margin as well as through the gingival sulci of the teeth being augmented. A fulcrum maneuver was then done to completely dissect the attached gingiva and allow for low-tension coronal repositioning of the gingiva (Figure 8). Once the dissection was complete in the A and B zones, the subperiosteal tunnel was extended into the C zone interproximally under each papilla, without making any surface incisions through the papilla.

**Figure 9:**
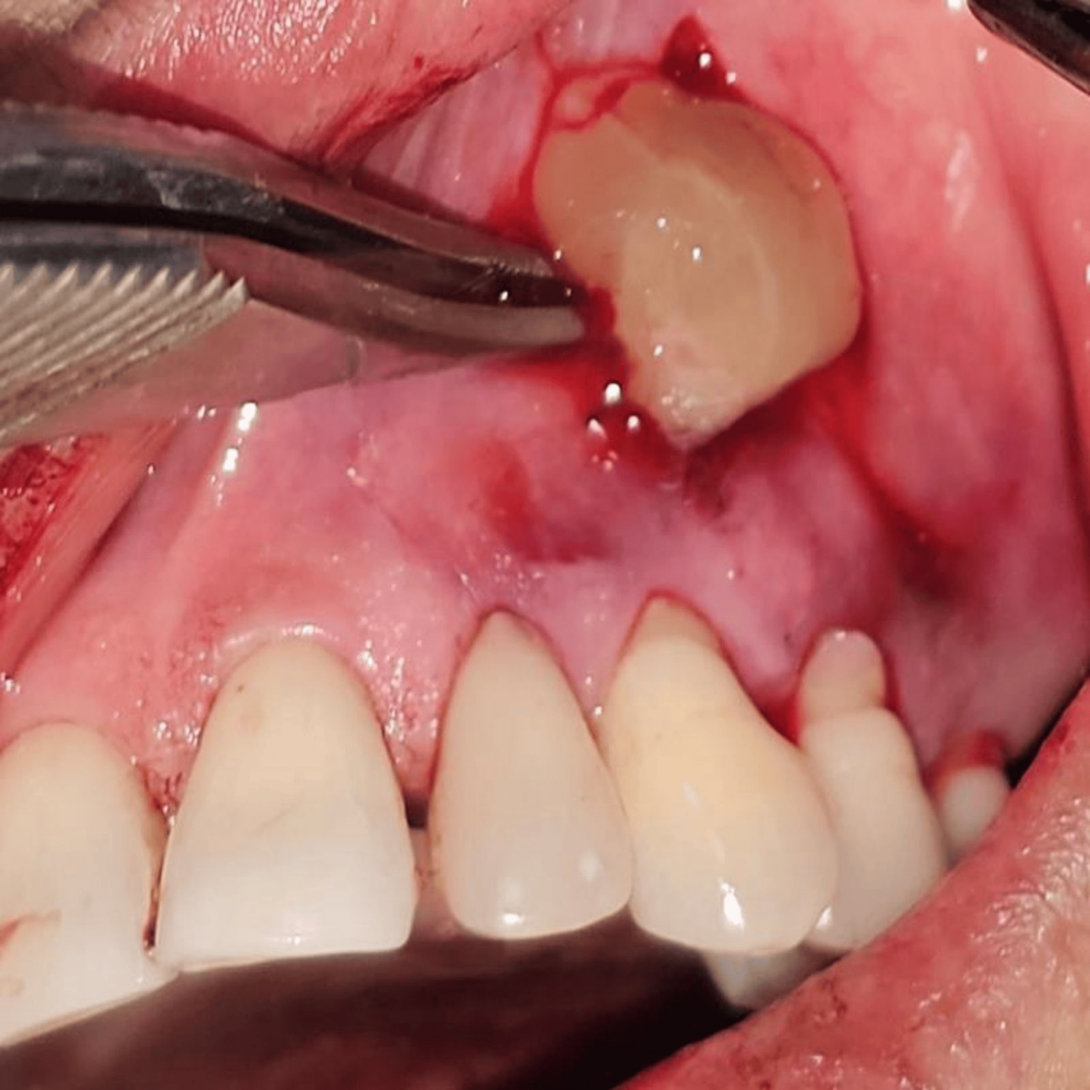
Placement of PRF inside the subperiosteal tunnel through vertical incision PRF: Platelet-rich fibrin

In Group I, intravenous blood was drawn in a 10-mL glass-coated plastic tube without the use of an anticoagulant before surgery. The blood was immediately centrifuged (3,000 revolutions, RCF 400 g, 10 min, and fixed centrifugation) under the standard protocol explained by Dohan and Choukroun [12] using a tabletop centrifugation machine (The Duo Quattro™ Advanced PRF Centrifuge). The obtained L-PRF was then separated from the red corpuscles base (preserving a small red blood cell layer) using sterile tweezers and scissors just after removal of platelet-poor plasma and put in the subperiosteal tunnel at the desired site with the help of fine-tipped curved serrated forceps (Figure 9).

**Figure 10:**
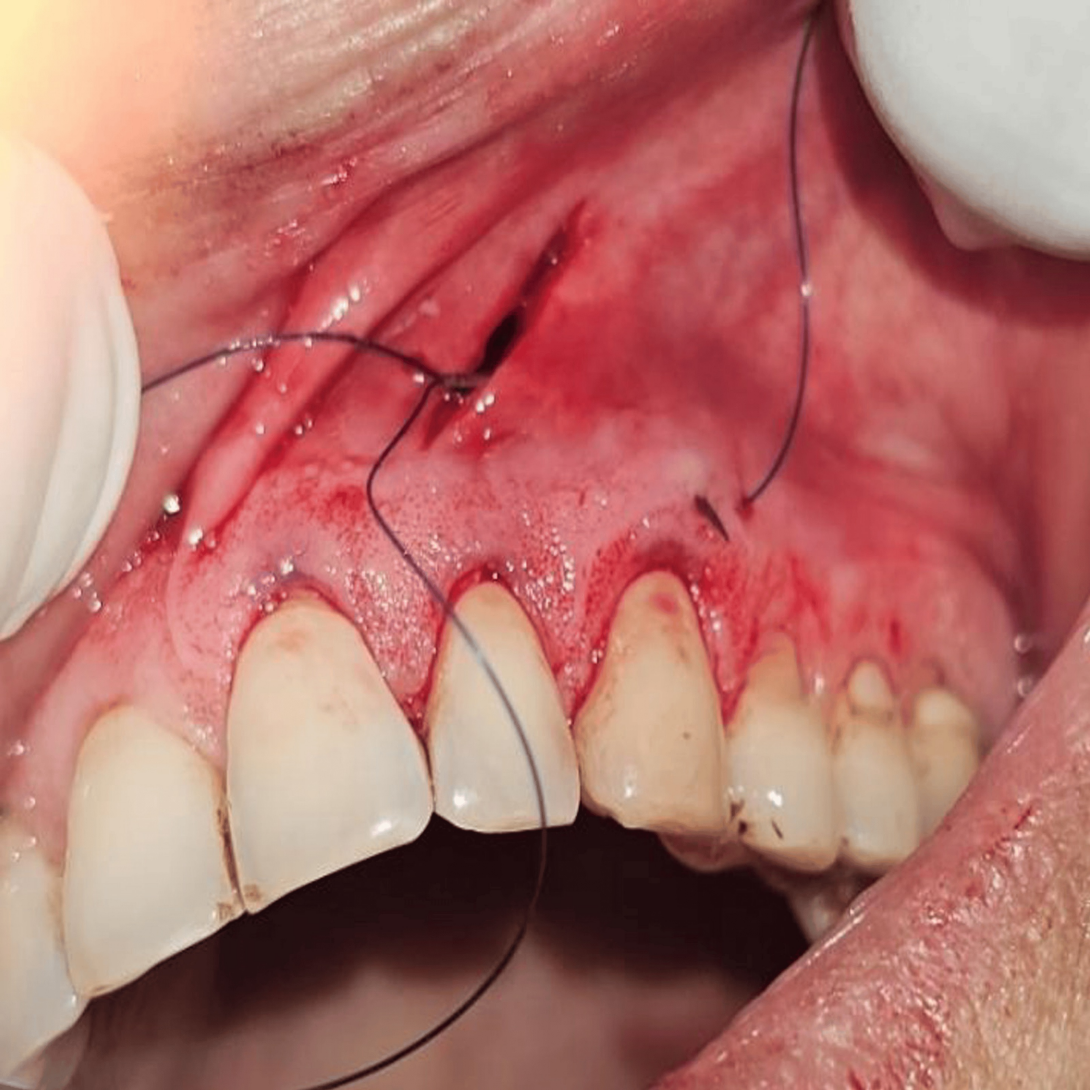
The Lasso sutures are used for guiding the amniotic membrane into the subperiosteal tunnel

**Figure 11:**
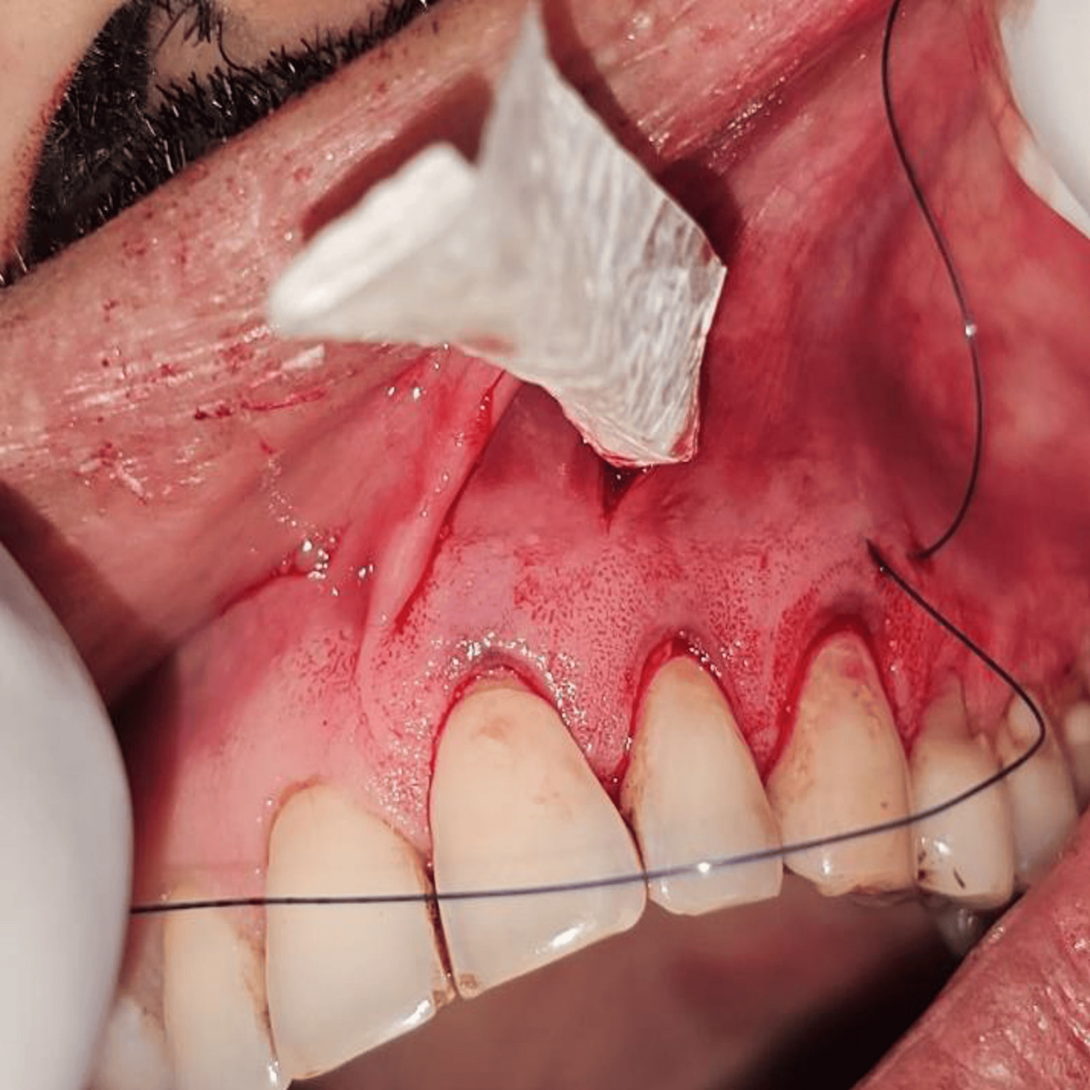
The sutures were passed through the AM and returned through the same path of entry to exit from the distal tooth sulcus AM: Amniotic membrane

In Group II, the recession defects were treated with freeze‑dried, irradiated amniotic membrane, which was procured from the tissue bank of Tata Memorial Hospital, Mumbai. The membrane was guided using a lasso suturing technique within the tunnel by inserting 4.0 Prolene sutures with a 22 mm 3/8 circle needle subperiosteally within the gingival sulcus of the distal most tooth and exiting through the vestibular access incision. The suture was then passed through the edge of the AM and returned through the same path of entry to exit from the distal tooth sulcus (Figures 10, 11). After placing either of the membranes, the tunnel and the entire mucogingival apparatus were then advanced coronally and stabilized in the new position using the double mattress coronally anchored suturing technique.

**Figure 12:**
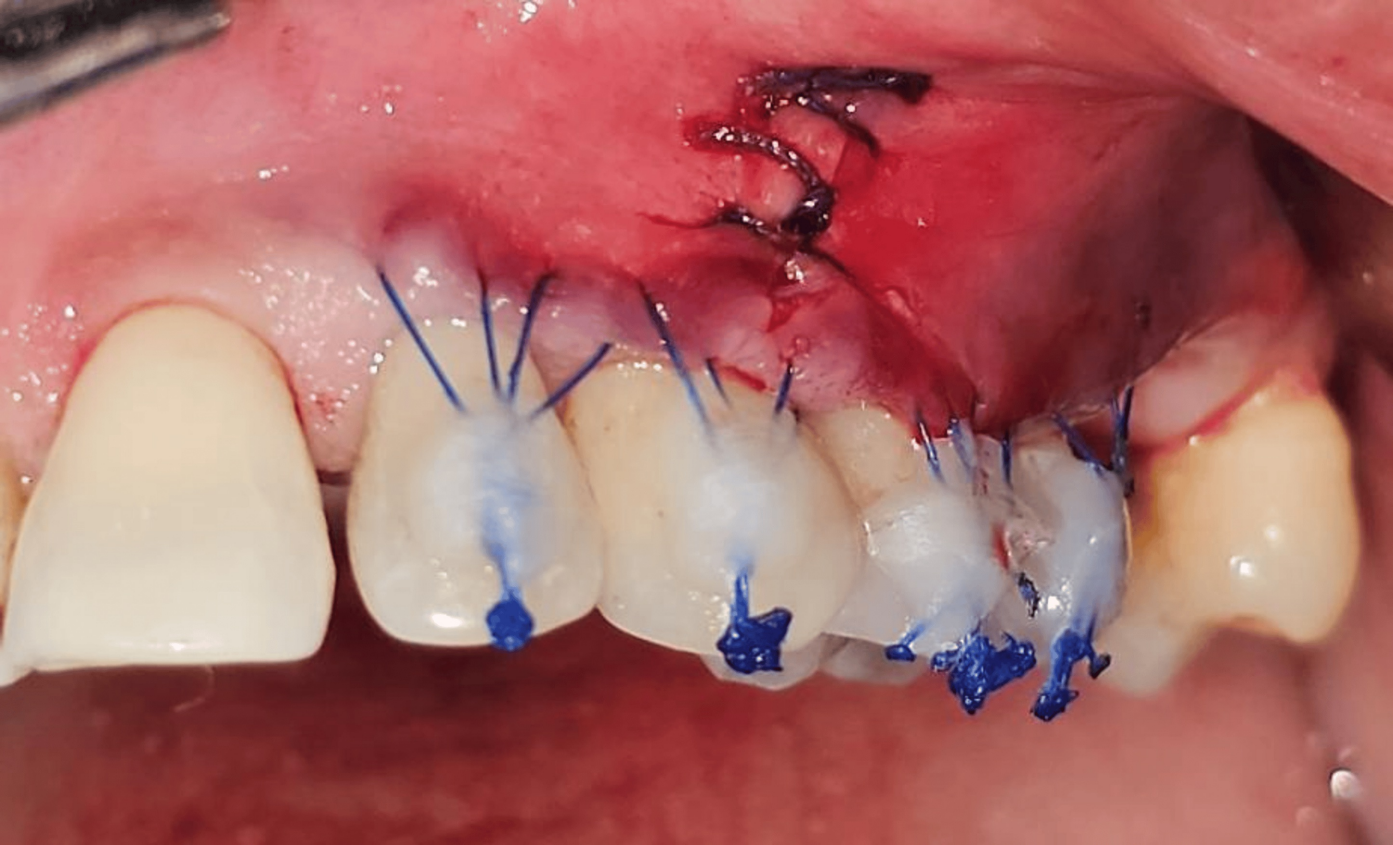
Double mattress coronally anchored sutures placed to move the gingival margin coronally. Vertical incision closed with internal mattress sutures

A specialized double mattress coronally anchored suturing technique is used with VISTA, which was the same for both Group I and Group II. A double mattress suture was given spanning the width of the defect. The technique consists of giving two horizontal mattress sutures at the mesial and distal sides of the tooth being treated, and about 2-3 mm apical to the gingival margin of each tooth. This suturing technique divides the tension equally at four points around each defect while coronally advancing the gingival margin, thus immobilizing the margin effectively during the healing period. The two ends of the suture are then advanced coronally and bonded to the composite stops placed on the teeth. This firm fixation of GM diminishes micromotion, thus providing a more promising outcome. The double mattress coronally anchored sutures also act as a stopper to avert the apical movement of the GM during the initial stages of healing. The vertical access incision is then closed with an internal mattress suture (Figure 12).

Postoperative care

Patients were prescribed antibiotics (Amoxicillin 500 mg, thrice daily for five days) and analgesics (Diclofenac sodium 50 mg, twice daily for three days), with instructions to use a 0.2% chlorhexidine mouth rinse twice daily for 10 days, starting 24 hours after the surgery. Participants were monitored for intraoperative and postoperative complications throughout the study period. Mild localized swelling at the operated site was observed in some participants during the early postoperative phase; however, the swelling was non-tender, self-limiting, and resolved without intervention. No other adverse events or unintended effects were reported in either study group during the follow-up period. Sutures from the vestibular access incision were removed after 10 days, and coronally anchored sutures were removed during the three-week follow-up to ensure proper immobilization during healing.

Follow-up

**Figure 13:**
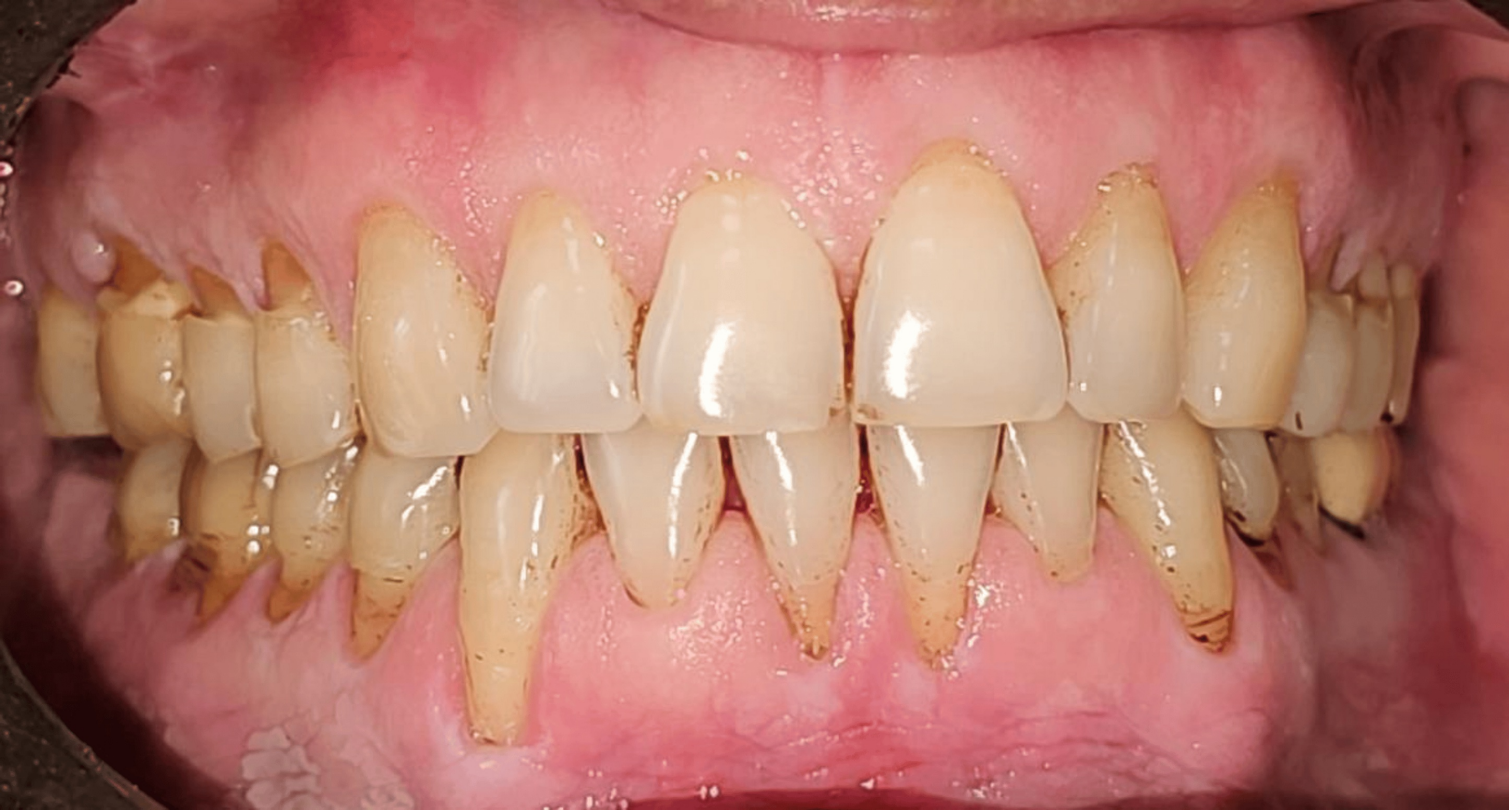
Pre-op view. Recessions are present on upper and lower incisors, canines, premolars, and molars on the left and right sides

**Figure 14:**
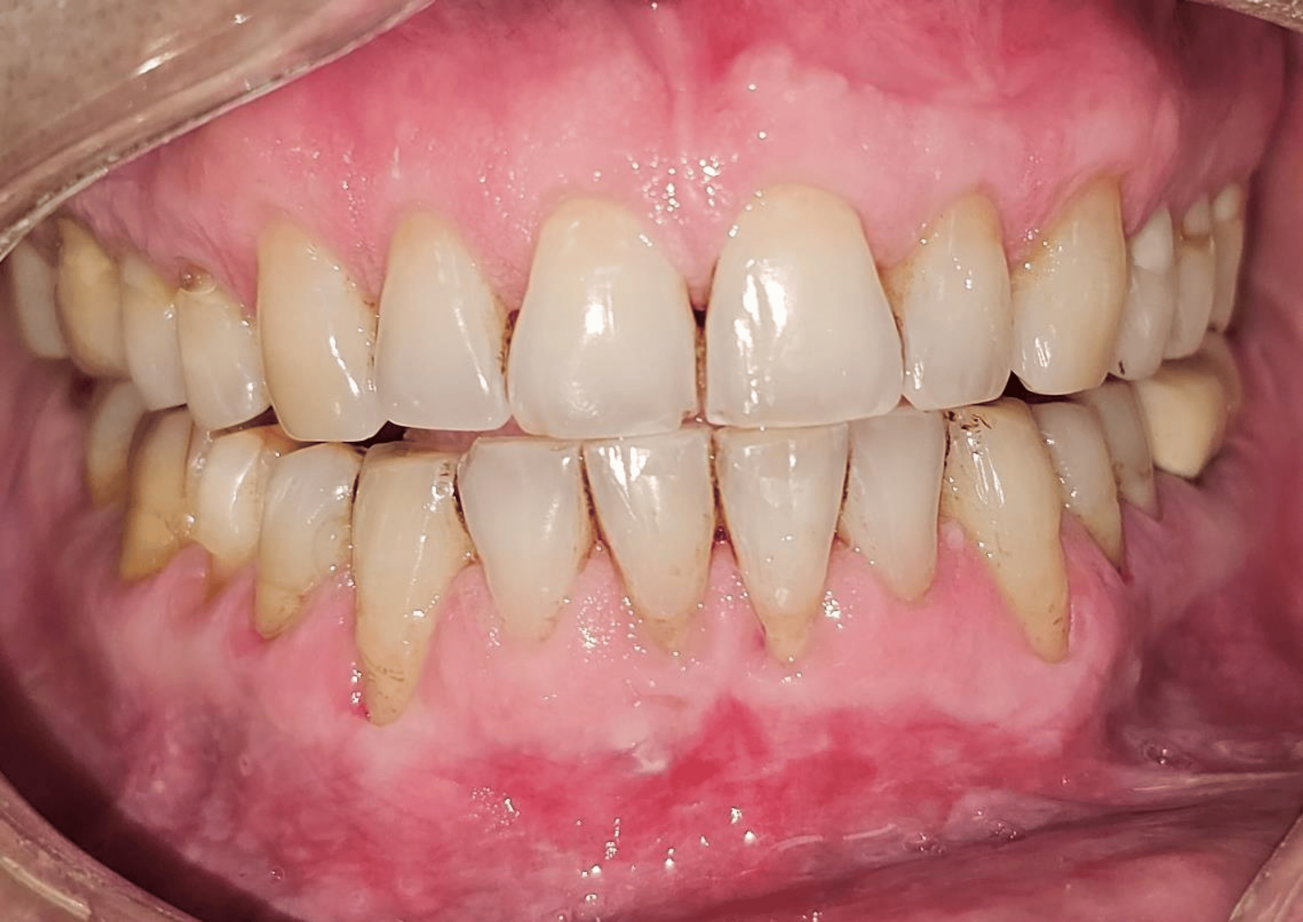
Post-op three-month follow-up. Significant coverage has been achieved in all the treated sites

**Figure 15:**
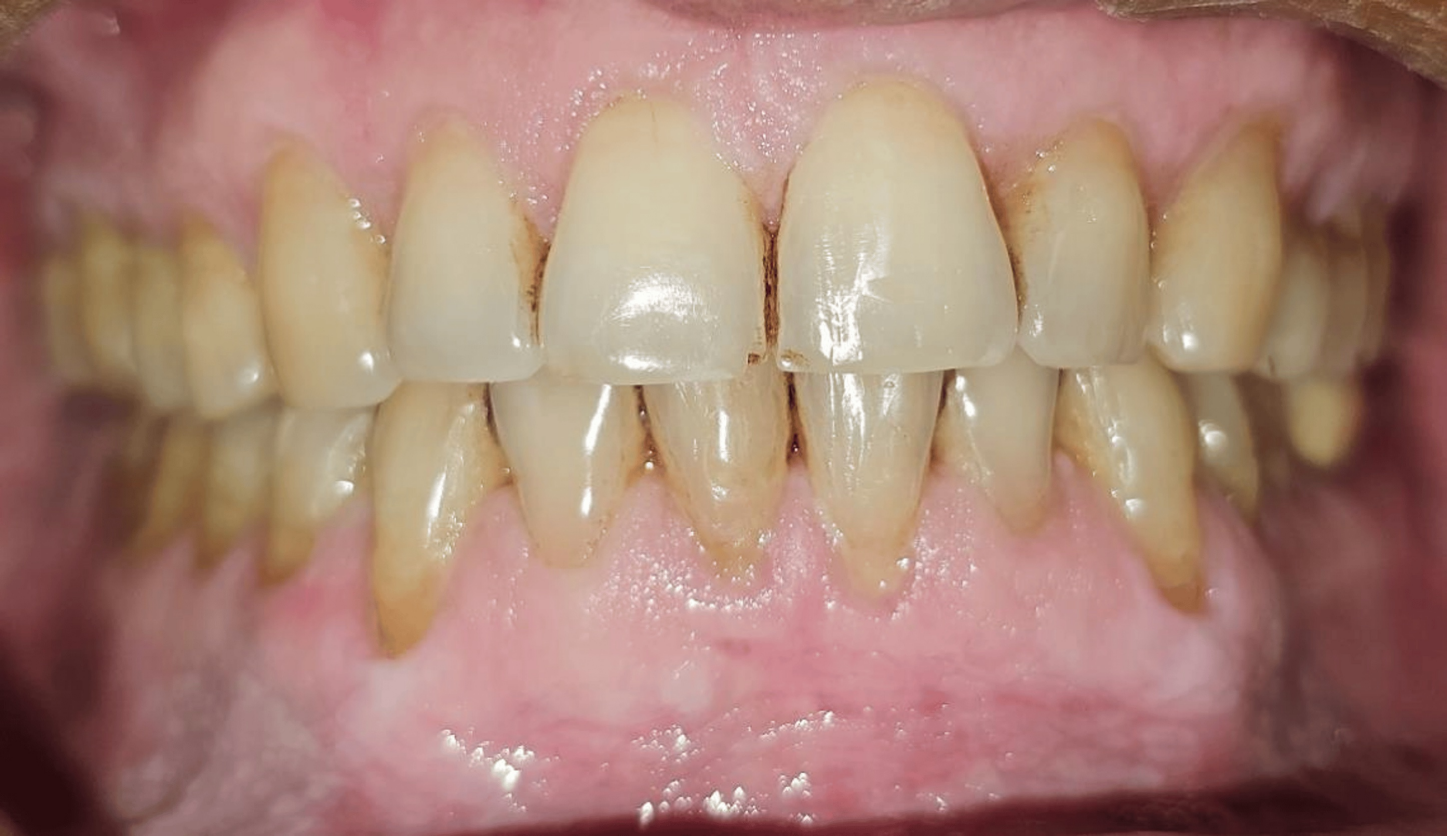
The six-month post-op follow-up shows stable soft tissue architecture and creeping attachment that has taken place on the lower left and right canines

Clinical measurements were recorded at baseline, and one, three, and six months postoperatively to assess the outcomes of the interventions in both groups (Figures 13-15).

Statistics

Data were coded and recorded in the MS Excel spreadsheet program. IBM Corp. Released 2017. IBM SPSS Statistics for Windows, Version 23. Armonk, NY: IBM Corp. was used for data analysis. Descriptive statistics were elaborated in the form of means/standard deviations and medians/IQRs for continuous variables and frequencies and percentages for categorical variables. The distribution of continuous variables was assessed using the Shapiro-Wilk test prior to statistical analysis. As the data did not follow a normal distribution (p < 0.05), nonparametric tests were employed. Accordingly, intergroup comparisons were performed using the Mann-Whitney U test, and intragroup comparisons across time points were analyzed using the Friedman test. Statistical significance was kept at p < 0.05. 

## Results

A total of seven patients (three males and four females) with a mean age of 42.00 ± 13.45 years, contributing to a sample size of 62 recession sites that met the inclusion criteria of the study, underwent root coverage surgery. No adverse events, such as inflammation, infection, or swelling, were reported during the healing period in both groups. A summary of all the clinical parameters is presented in Table 1.

**Table 1:** Comparison of mean values of the clinical parameters over time ᵗ = t test, ᵐ = Mann-Whitney U Test, ᶠ = Fisher's Exact Test, PPD: Pocket probing depth, MRC: Mean root coverage, WKG: Width of keratinized gingiva, RAL: Relative attachment level, RW: Recession width, RH: Recession height

Variable	Parameter	Total	Group		Difference (95% CI)	Significance
			I	II		
PPD (Baseline)	Mean ± SD	2.13 ± 0.34	2.13 ± 0.34	2.13 ± 0.34	0.00 (-0.17 to 0.17)	W = 480.500, p = 1.000ᵐ
	Median (IQR)	2.00 (2.00 - 2.00)	2.00 (2.00 - 2.00)	2.00 (2.00 - 2.00)		
PPD (One Month)	Mean ± SD	2.37 ± 1.27	3.55 ± 0.51	1.19 ± 0.40	2.35 (2.12 to 2.59)	W = 961.000, p = <0.001ᵐ
	Median (IQR)	2.50 (1.00 - 4.00)	4.00 (3.00 - 4.00)	1.00 (1.00 - 1.00)		
PPD (Three Months)	Mean ± SD	2.10 ± 1.10	3.10 ± 0.54	1.10 ± 0.30	2.00 (1.78 to 2.22)	W = 956.500, p = <0.001ᵐ
	Median (IQR)	2.00 (1.00 - 3.00)	3.00 (3.00 - 3.00)	1.00 (1.00 - 1.00)		
PPD (Six Months)	Mean ± SD	1.23 ± 0.40	1.35 ± 0.45	1.10 ± 0.30	0.26 (0.06 to 0.45)	W = 629.500, p = 0.006ᵐ
	Median (IQR)	1.00 (1.00 - 2.00)	1.00 (1.00 - 2.00)	1.00 (1.00 - 1.00)		
RH (Baseline)	Mean ± SD	2.64 ± 0.97	2.63 ± 0.89	2.65 ± 1.05	-0.02 (-0.51 to 0.48)	W = 468.500, p = 0.862ᵐ
	Median (IQR)	3.00 (2.00 - 3.00)	2.00 (2.00 - 3.00)	3.00 (2.00 - 3.00)		
RH (One Month)	Mean ± SD	1.53 ± 0.63	1.53 ± 0.63	1.53 ± 0.64	0.00 (-0.32 to 0.32)	W = 477.000, p = 0.965ᵐ
	Median (IQR)	1.50 (1.00 - 2.00)	1.50 (1.00 - 2.00)	1.50 (1.00 - 2.00)		
RH (Three Months)	Mean ± SD	1.15 ± 0.62	1.19 ± 0.67	1.11 ± 0.57	0.08 (-0.24 to 0.40)	W = 494.500, p = 0.843ᵐ
	Median (IQR)	1.00 (1.00 - 2.00)	1.00 (0.50 - 2.00)	1.00 (1.00 - 1.50)		
RH (Six Months)	Mean ± SD	0.96 ± 0.49	0.97 ± 0.48	0.95 ± 0.51	0.02 (-0.23 to 0.27)	W = 486.000, p = 0.940ᵐ
	Median (IQR)	1.00 (0.50 - 1.50)	1.00 (0.50 - 1.50)	1.00 (1.00 - 1.00)		
RW (Baseline)	Mean ± SD	2.76 ± 1.25	3.19 ± 1.33	2.32 ± 1.01	0.87 (0.27 to 1.47)	W = 684.000, p = 0.003ᵐ
	Median (IQR)	2.00 (2.00 - 3.50)	3.00 (2.00 - 3.50)	2.00 (2.00 - 3.00)		
RW (One Month)	Mean ± SD	1.66 ± 1.00	2.05 ± 1.16	1.27 ± 0.62	0.77 (0.30 to 1.25)	W = 690.000, p = 0.002ᵐ
	Median (IQR)	1.25 (1.00 - 2.50)	2.00 (1.00 - 2.50)	1.00 (1.00 - 2.00)		
RW (Three Months)	Mean ± SD	1.25 ± 0.86	1.60 ± 0.94	0.90 ± 0.60	0.69 (0.29 to 1.10)	W = 702.000, p = 0.001ᵐ
	Median (IQR)	1.00 (0.62 - 2.00)	1.00 (1.00 - 2.00)	1.00 (0.50 - 1.50)		
RW (Six Months)	Mean ± SD	1.04 ± 0.81	1.32 ± 0.94	0.76 ± 0.53	0.56 (0.17 to 0.96)	W = 675.500, p = 0.005ᵐ
	Median (IQR)	1.00 (0.50 - 2.00)	1.00 (0.50 - 2.00)	0.50 (0.50 - 1.00)		
RAL (Baseline)	Mean ± SD	10.35 ± 1.87	10.06 ± 2.06	10.65 ± 1.62	-0.58 (-1.53 to 0.36)	W = 407.500, p = 0.287ᵐ
	Median (IQR)	11.00 (9.00 - 12.00)	10.00 (9.00 - 12.00)	12.00 (9.50 - 12.00)		
RAL (One Month)	Mean ± SD	9.39 ± 1.88	9.06 ± 2.06	9.71 ± 1.66	-0.65 (-1.60 to 0.31)	W = 398.000, p = 0.231ᵐ
	Median (IQR)	10.00 (8.00 - 11.00)	9.00 (8.00 - 11.00)	11.00 (8.50 - 11.00)		
RAL (Three Months)	Mean ± SD	9.34 ± 1.93	9.06 ± 2.06	9.61 ± 1.78	-0.55 (-1.53 to 0.43)	W = 408.500, p = 0.295ᵐ
	Median (IQR)	10.00 (8.00 - 11.00)	9.00 (8.00 - 11.00)	11.00 (8.50 - 11.00)		
RAL (Six Months)	Mean ± SD	9.34 ± 1.93	9.06 ± 2.06	9.61 ± 1.78		
	Median (IQR)	10.00 (8.00 - 11.00)	9.00 (8.00 - 11.00)	11.00 (8.50 - 11.00)		
WKG (Baseline)	Mean ± SD	1.57 ± 0.49	1.52 ± 0.47	1.63 ± 0.52	-0.11 (-0.36 to 0.14)	W = 420.000, p = 0.376ᵐ
	Median (IQR)	1.50 (1.00 - 2.00)	1.50 (1.00 - 2.00)	1.50 (1.00 - 2.00)		
WKG (One Month)	Mean ± SD	2.27 ± 0.47	2.19 ± 0.36	2.35 ± 0.55	-0.16 (-0.40 to 0.08)	W = 382.000, p = 0.148ᵐ
	Median (IQR)	2.25 (2.00 - 2.50)	2.00 (2.00 - 2.50)	2.50 (2.00 - 3.00)		
WKG (Three Months)	Mean ± SD	2.27 ± 0.47	2.19 ± 0.36	2.35 ± 0.55		
	Median (IQR)	2.25 (2.00 - 2.50)	2.00 (2.00 - 2.50)	2.50 (2.00 - 3.00)		
WKG (Six Months)	Mean ± SD	2.27 ± 0.47	2.19 ± 0.36	2.35 ± 0.55		
	Median (IQR)	2.25 (2.00 - 2.50)	2.00 (2.00 - 2.50)	2.50 (2.00 - 3.00)		
MRC (%)	Mean ± SD	64.91 ± 15.21	64.44 ± 13.88	65.38 ± 16.66	-0.94 (-8.74 to 6.85)	W = 489.500, p = 0.901ᵐ
	Median (IQR)	66.67 (50.00 - 75.00)	66.67 (50.00 - 75.00)	66.67 (50.00 - 66.67)		

Pocket probing depth (PPD) showed significant changes over time in both groups (Table 2). In Group I, the mean PPD increased from 2.13 at baseline to 3.55 at one month, followed by a progressive reduction to 1.35 at six months; this intra-group change was statistically significant (Friedman test: χ² = 86.0, p < 0.001). In Group II, mean PPD demonstrated a continuous reduction from 2.13 at baseline to 1.10 at six months, with the change also being statistically significant (Friedman test: χ² = 84.8, p < 0.001). Intergroup comparison revealed no significant difference in PPD at baseline (W = 480.500, p = 1.000). However, significant differences between the two groups were observed at one month (W = 961.000, p < 0.001), three months (W = 956.500, p < 0.001), and six months (W = 629.500, p = 0.006).

**Table 2:** Comparison of the two groups in terms of change in PPD over time PPD: Pocket probing depth Non-parametric tests were used to make statistical inferences as data were not normally distributed. The Wilcoxon-Mann-Whitney test was used to compare the two groups in terms of PPD at each of the time points. The Friedman test was used to explore the change in PPD over time within each group. The Generalized Estimating Equations method was used to explore the difference in change in PPD between the two groups over time. There was no significant difference between the groups in terms of PPD (Baseline) (W = 480.500, p = 1.000). There was a significant difference between the 2 groups in terms of PPD (one month) (W = 961.000, p = <0.001). There was a significant difference between the 2 groups in terms of PPD (three months) (W = 956.500, p = <0.001). There was a significant difference between the 2 groups in terms of PPD (six months) (W = 629.500, p = 0.006). The two groups differed significantly in terms of PPD at the following timepoints: one month, three months, and six months. In Group I, the mean PPD increased from 2.13 at the baseline timepoint to a maximum of 3.55 at the one-month timepoint and then decreased to 1.35 at the six-month timepoint. This change was statistically significant (Friedman Test: χ2 = 86.0, p = <0.001). In Group II, the mean PPD decreased from a maximum of 2.13 at the baseline timepoint to a minimum of 1.10 at the six-month timepoint. This change was statistically significant (Friedman Test: χ2 = 84.8, p = <0.001). The overall change in PPD over time was compared in the two groups using the Generalized Estimating Equations method. There was a significant difference in the trend of PPD over time between the two groups (p = <0.001); *p < 0.05 was considered statistically significant.

PPD	Group I Mean (SD)	Group II Mean (SD)	W value	p-value for comparison of the two groups at each of the timepoints (Wilcoxon-Mann-Whitney Test)
Baseline	2.13 (0.34)	2.13 (0.34)	480.500	1.000
One Month	3.55 (0.51)	1.19 (0.40)	961.000	<0.001
Three Months	3.10 (0.54)	1.10 (0.30)	956.500	<0.001
Six Months	1.35 (0.45)	1.10 (0.30)	629.500	0.006
Friedman Test: χ2	86	84.8		
p-value for change in PPD over time within each group (Friedman Test)	<0.001	<0.001		
Overall p-value for comparison of change in PPD over time between the two groups (Generalized Estimating Equations)	<0.001			

Recession height (RH) demonstrated a significant reduction over time in both groups (Table 3). Intergroup comparison revealed no statistically significant difference in RH at any of the evaluated timepoints, including baseline (W = 468.500, p = 0.862), one month (W = 477.000, p = 0.965), three months (W = 494.500, p = 0.843), and six months (W = 486.000, p = 0.940). In Group I, the mean RH decreased from 2.63 at baseline to 0.97 at six months, and this intra-group change was statistically significant (Friedman test: χ² = 83.8, p < 0.001). Similarly, Group II showed a significant reduction in mean RH from 2.65 at baseline to 0.95 at six months (Friedman test: χ² = 84.0, p < 0.001).

**Table 3:** Comparison of the two groups in terms of change in RH over time RH: Recession height, PPD: Pocket probing depth Non-parametric tests were used to make statistical inferences as data were not normally distributed. The Wilcoxon-Mann-Whitney test was used to compare the two groups in terms of RH at each of the timepoints. The Friedman test was used to explore the change in RH over time within each group. The Generalized Estimating Equations method was used to explore the difference in change in RH between the two groups over time. There was no significant difference between the groups in terms of RH (Baseline) (W = 468.500, p = 0.862). There was no significant difference between the groups in terms of RH (one Month) (W = 477.000, p = 0.965). There was no significant difference between the groups in terms of RH (three months) (W = 494.500, p = 0.843). There was no significant difference between the groups in terms of RH (six months) (W = 486.000, p = 0.940). The two groups did not differ in terms of RH at any of the timepoints. In Group I, the mean RH decreased from a maximum of 2.63 at the baseline timepoint to a minimum of 0.97 at the six-month timepoint. This change was statistically significant (Friedman Test: χ² = 83.8, p = <0.001). In Group II, the mean RH decreased from a maximum of 2.65 at the baseline timepoint to a minimum of 0.95 at the six-month timepoint. This change was statistically significant (Friedman Test: χ2 = 84.0, p = <0.001). The overall change in RH over time was compared in the two groups using the Generalized Estimating Equations method. There was no significant difference in the trend of RH over time between the two groups (p = 0.703); *p < 0.05 was considered statistically significant.

RH	Group I Mean (SD)	Group II Mean (SD)	W value	p-value for comparison of the two groups at each of the timepoints (Wilcoxon-Mann-Whitney Test)
Baseline	2.63 (0.89)	2.65 (1.05)	468.500	0.862
One Month	1.53 (0.63)	1.53 (0.64)	477.000	0.965
Three Months	1.19 (0.67)	1.11 (0.57)	494.500	0.843
Six Months	0.97 (0.48)	0.95 (0.51)	486.000	0.940
Friedman Test: χ2	83.8	84.00		
p-value for change in PPD over time within each group (Friedman Test)	<0.001	<0.001		
Overall p-value for comparison of change in PPD over time between the two groups (Generalized Estimating Equations)	0.703			

Recession width (RW) showed a significant reduction over time in both groups (Table 4). Intergroup comparison revealed statistically significant differences in RW at all evaluated timepoints, including baseline (W = 684.000, p = 0.003), one month (W = 690.000, p = 0.002), three months (W = 702.000, p = 0.001), and six months (W = 675.500, p = 0.005). In Group I, the mean RW decreased from 3.19 at baseline to 1.32 at six months, and this intra-group change was statistically significant (Friedman test: χ² = 82.8, p < 0.001). Similarly, Group II demonstrated a significant reduction in mean RW from 2.32 at baseline to 0.76 at six months (Friedman test: χ² = 81.1, p < 0.001).

**Table 4:** Comparison of the two groups in terms of change in RW over time PPD: Pocket probing depth Non-parametric tests were used to make statistical inferences as data were not normally distributed. The Wilcoxon-Mann-Whitney test was used to compare the two groups in terms of RW at each of the timepoints. The Friedman test was used to explore the change in RW over time within each group. The Generalized Estimating Equations method was used to explore the difference in change in RW between the two groups over time. There was a significant difference between the 2 groups in terms of RW (Baseline) (W = 684.000, p = 0.003). There was a significant difference between the 2 groups in terms of RW (one month) (W = 690.000, p = 0.002). There was a significant difference between the 2 groups in terms of RW (three months) (W = 702.000, p = 0.001). There was a significant difference between the 2 groups in terms of RW (six months) (W = 675.500, p = 0.005). The two groups differed significantly in terms of RW at the following timepoints: baseline, one month, three months, and six months. In Group I, the mean RW decreased from a maximum of 3.19 at the baseline timepoint to a minimum of 1.32 at the six-month timepoint. This change was statistically significant (Friedman Test: χ2 = 82.8, p = <0.001). In Group II, the mean RW decreased from a maximum of 2.32 at the baseline timepoint to a minimum of 0.76 at the six-month timepoint. This change was statistically significant (Friedman Test: χ2 = 81.1, p = <0.001). The overall change in RW over time was compared in the two groups using the Generalized Estimating Equations method. There was no significant difference in the trend of RW over time between the two groups (p = 0.245); *p < 0.05 was considered statistically significant.

RW	Group I Mean (SD)	Group II Mean (SD)	W value	p-value for comparison of the two groups at each of the timepoints (Wilcoxon-Mann-Whitney Test)
Baseline	3.19 (1.33)	2.32 (1.01)	684.000	0.003
One Month	2.05 (1.16)	1.27 (0.62)	690.000	0.002
Three Months	1.60 (0.94)	0.90 (0.60)	702.000	0.001
Six Months	1.32 (0.94)	0.76 (0.53)	675.500	0.005
Friedman Test: χ2	82.8	81.1		
p-value for change in PPD over time within each group (Friedman Test)	<0.001	<0.001		
Overall p-value for comparison of change in PPD over time between the two groups (Generalized Estimating Equations)	0.245			

Relative attachment level (RAL) demonstrated a significant reduction over time in both groups (Table 5). Intergroup comparison revealed no statistically significant differences in RAL at any of the evaluated timepoints, including baseline (W = 407.500, p = 0.287), one month (W = 398.000, p = 0.231), three months (W = 408.500, p = 0.295), and six months (W = 408.500, p = 0.295). In Group I, the mean RAL decreased from 10.06 at baseline to 9.06 at six months, with this intra-group change being statistically significant (Friedman test: χ² = 93.0, p < 0.001). Similarly, Group II showed a significant reduction in mean RAL from 10.65 at baseline to 9.61 at six months (Friedman test: χ² = 84.8, p < 0.001).

**Table 5:** Comparison of the two groups in terms of change in RAL over time RAL: Relative attachment level, PPD: Pocket probing depth Non-parametric tests were used to make statistical inferences, as data were not normally distributed. The Wilcoxon-Mann-Whitney test was used to compare the two groups in terms of RAL at each of the timepoints. The Friedman test was used to explore the change in RAL over time within each group. The Generalized Estimating Equations method was used to explore the difference in change in RAL between the two groups over time. There was no significant difference between the groups in terms of RAL (Baseline) (W = 407.500, p = 0.287). There was no significant difference between the groups in terms of RAL (one month) (W = 398.000, p = 0.231). There was no significant difference between the groups in terms of RAL (three months) (W = 408.500, p = 0.295). There was no significant difference between the groups in terms of RAL (six months) (W = 408.500, p = 0.295). The two groups did not differ in terms of RAL at any of the timepoints. In Group I, the mean RAL decreased from a maximum of 10.06 at the baseline timepoint to a minimum of 9.06 at the six-month timepoint. This change was statistically significant (Friedman Test: χ2 = 93.0, p = <0.001). In Group II, the mean RAL decreased from a maximum of 10.65 at the baseline timepoint to a minimum of 9.61 at the six-month timepoint. This change was statistically significant (Friedman Test: χ2 = 84.8, p = <0.001). The overall change in RAL over time was compared in the two groups using the Generalized Estimating Equations method. There was no significant difference in the trend of RAL over time between the two groups (p = -); *p < 0.05 was considered statistically significant.

RAL	Group I Mean (SD)	Group II Mean (SD)	W value	p-value for comparison of the two groups at each of the timepoints (Wilcoxon-Mann-Whitney Test)
Baseline	10.06 (2.06)	10.65 (1.62)	407.500	0.287
One Month	9.06 (2.06)	9.71 (1.66)	398.000	0.231
Three Months	9.06 (2.06)	9.61 (1.78)	408.500	0.295
Six Months	9.06 (2.06)	9.61 (1.78)	408.500	0.295
Friedman Test: χ2	93.0	84.8		
p-value for change in PPD over time within each group (Friedman Test)	<0.001	<0.001		
Overall p-value for comparison of change in PPD over time between the two groups (Generalized Estimating Equations)	-			

The width of keratinized gingiva (WKG) showed a significant increase over time in both groups (Table 6). Intergroup comparison revealed no statistically significant differences in WKG at any of the evaluated timepoints, including baseline (W = 420.000, p = 0.376), one month (W = 382.000, p = 0.148), three months (W = 382.000, p = 0.148), and six months (W = 382.000, p = 0.148). In Group I, the mean WKG increased from 1.52 at baseline to 2.19 at six months, and this intra-group change was statistically significant (Friedman test: χ² = 87.0, p < 0.001). Similarly, Group II demonstrated a significant increase in mean WKG from 1.63 at baseline to 2.35 at six months (Friedman test: χ² = 93.0, p < 0.001). Although Group II showed a numerically greater increase in WKG, the difference between the two groups was not statistically significant.

**Table 6:** Comparison of the two groups in terms of change in WKG over time WKG: Width of keratinized gingiva, PPD: Pocket probing depth Non-parametric tests were used to make statistical inferences as data were not normally distributed. The Wilcoxon-Mann-Whitney test was used to compare the two groups in terms of WKG at each of the time points. The Friedman test was used to explore the change in WKG over time within each group. The Generalized Estimating Equations method was used to explore the difference in change in WKG between the two groups over time. There was no significant difference between the groups in terms of WKG (baseline) (W = 420.000, p = 0.376). There was no significant difference between the groups in terms of WKG (one month) (W = 382.000, p = 0.148). There was no significant difference between the groups in terms of WKG (three months) (W = 382.000, p = 0.148). There was no significant difference between the groups in terms of WKG (six months) (W = 382.000, p = 0.148). The two groups did not differ in terms of WKG at any of the timepoints. In Group I, the mean WKG increased from a minimum of 1.52 at the baseline timepoint to a maximum of 2.19 at the six-month timepoint. This change was statistically significant (Friedman Test: χ2 = 87.0, p = <0.001). In Group II, the mean WKG increased from a minimum of 1.63 at the baseline timepoint to a maximum of 2.35 at the six-month timepoint. This change was statistically significant (Friedman Test: χ2 = 93.0, p = <0.001). The overall change in WKG overtime was compared in the two groups using the generalized estimating equations method. There was no significant difference in the trend of WKG over time between the two groups (p = -); *p < 0.05 was considered statistically significant.

WKG	Group I Mean (SD)	Group II Mean (SD)	W value	p-value for comparison of the two groups at each of the timepoints (Wilcoxon-Mann-Whitney Test)
Baseline	1.52 (0.47)	1.63 (0.52)	420.000	0.376
One Month	2.19 (0.36)	2.35 (0.55)	382.000	0.148
Three Months	2.19 (0.36)	2.35 (0.55)	382.000	0.148
Six Months	2.19 (0.36)	2.35 (0.55)	382.000	0.148
Friedman Test: χ2	87.00	93.00		
p-value for change in PPD over time within each group (Friedman Test)	<0.001	<0.001		
Overall p-value for comparison of change in PPD over time between the two groups (Generalized Estimating Equations)	-			

The mean (SD) of mean root coverage (MRC) was 64.44% (13.88) in Group I and 65.38% (16.66) in Group II (Table 7). The median (IQR) MRC was 66.67% (50-75) for Group I and 66.67% (50-66.67) for Group II. In both groups, MRC values ranged from 50% to 100%. Intergroup comparison revealed no statistically significant difference in MRC between the two groups (W = 489.500, p = 0.901).

**Table 7:** Association between ‘Group’ and ‘MRC (%)’ MRC: Mean root coverage The variable MRC (%) was not normally distributed in the two subgroups of the variable Group. Thus, non-parametric tests (Wilcoxon-Mann-Whitney U Test) were used to make group comparisons. The median (IQR) of MRC (%) in the Group is as follows: Gorup I was 66.67 (50-75). The median (IQR) of MRC (%) in the Group II was 66.67 (50-66.67). There was no significant difference between the groups in terms of MRC (%) (W = 489.500, p = 0.901)

MRC (%)	Group		Wilcoxon-Mann-Whitney U Test	
	I	II	W	p-value
Mean (SD)	64.44 (13.88)	65.38 (16.66)	489.500	0.901
Median (IQR)	66.67 (50-75)	66.67 (50-66.67)		
Min - Max	50 - 100	50 - 100		

## Discussion

The study explores the effectiveness of vestibular incision subperiosteal tunnel access (VISTA) with platelet-rich fibrin (PRF) and amniotic membrane (AM) in treating gingival recession (GR). VISTA, a minimally invasive surgical approach developed by Dr. Zadeh in 2011, allows for coronal advancement of gingiva through a single incision, making it a favorable option for GR treatment [5].

Recession treatment poses challenges such as restoring aesthetic balance, regenerating periodontal support structures, and overcoming issues like avascular areas, large donor tissue requirements, and non-carious cervical lesions. These factors often lead to inadequate root coverage or recurrence due to muscle pull on healing tissues. VISTA addresses these challenges by preserving vascular supply and improving clinical outcomes.

In our study, the mean (SD) recession height (RH) in Group I decreased from 2.63 (0.89) at baseline to 0.97 (0.48) at the six-month time point. Similarly, the mean (SD) RH in Group II decreased from 2.65 (1.05) at baseline to 0.95 (0.51) at the six-month time point. The reduction in both groups from baseline to the three-month timepoint and from baseline to the six-month timepoint was statistically significant; however, intergroup comparison showed no statistical significance between the two groups. These results are identical to the observations made by Shetty SS et al. in their case report [13]. In a study by Esteves et al. [14], the application of AM for root coverage leads to a substantial decrease in recession depth (RD) from 3.14 ± 1.24 to 2.76 ± 1.00 (root coverage (RC) 22%). Similarly, Ankit A et al. [15] observed that when employed for root coverage, both AM and PRF demonstrated a reduction in RD with RC% (24% and 22%), respectively.

On comparing recession width (RW) in both groups, the mean (SD) RW in Group I decreased from 3.19 (1.33) at baseline to 1.32 (0.94) at the six-month time point. In Group II mean (SD), RW decreased from 2.32 (1.01) at baseline to 0.76 (0.53) at the six-month time point. The decrease in RW showed statistical significance in both groups over time, and statistical significance was observed in the intergroup comparison between the two groups.

Significant recession coverage was found by comparing the various time intervals of both groups. These findings are similar to research by Jankovic S et al. [8], who observed in a six-month randomized controlled clinical trial that PRF membrane provided clinically acceptable results and improved wound healing in comparison to recession sites that were treated with connective tissue graft. Reddy S et al. [15] also reported two cases in which PRF membrane was used with the modified coronally advanced flap (CAF) technique and demonstrated improved root coverage along with increased gingival thickness. Similarly, Padma R et al. [16] found that adding PRF to CAF resulted in superior root coverage. These findings may be explained by PRF's ability to gradually release cytokines during fibrin matrix remodeling during the healing process [17] and by the high percentage of undamaged platelets contained within a fibrin matrix. Additionally, the fibrin matrix in PRF serves as a fibrin glue, promoting neovascularization, decreasing necrosis, and keeping the flap in place [18].

The study observed a noteworthy enhancement in the recession coverage of Group II, which aligns with the findings of Gurinsky B [19]. Gurinsky B stated that AM can serve as a viable substitute for autogenous grafts, based on data gathered from a case series consisting of five instances. Similarly, after applying AM and connective tissue grafts (CTG) to 71 gingival recession sites, Ghahroudi AA et al. saw similar outcomes in terms of RC [20]. In their individual case reports, Shah R et al. and Mehta TN et al. noted improved wound healing and aesthetics with AM [18,21]. Mahajan R used AM on the guided tissue regeneration (GTR) principles and found it to be effective for gingival tissue augmentation due to its specific biological properties like resorbability, ability to mold according to defect morphology, and improved clinical handling [22]. These outcomes, in addition to the findings of the current investigation, can be ascribed to the induction of fibroblast proliferation and the presence of vascular growth factor in AM, which may quicken tissue maturation and angiogenesis while averting necrosis of the flap's coronal portion, promoting better healing and more creeping attachment [23].

The mean (SD) mean root coverage (MRC) (%) observed in Group I was 64.44 (13.88), while that in Group II was 65.38 (16.66). There was no statistical difference between the two groups; however, AM showed slightly better mean root coverage as compared to the PRF group. Agarwal et al. reported 56% MRC [24] with PRF-treated sites, but Jankovic et al. [8] reported 88.7%. However, investigations by Wallace [23] and Agarwal et al. [24] revealed 57% and 36%, respectively, when compared to the AM-treated location.

The pocket probing depth (PPD) values in Group I showed a significant increase at the three-month follow-up, which was followed by a significant reduction at six months as compared to baseline. Similar observations were made by Eren and Atilla [25], Jankovic et al. [8], and Aroca et al. [26] in their respective investigations. They concluded that the changes in PPD at six months were significant in the PRF group. Group II showed a significant reduction in the PPD values at the three- and six-month follow-up, compared to baseline. Wallace reported a 0.8 mm reduction in probing depth at four months using a placental-derived membrane, as compared to our investigation, which showed a 0.94 mm reduction [23].

Significant changes after the root coverage procedure were corroborated by the compatible condition of gingival health after initial therapy and patient selection by Paolantonio [27], thus reporting the expectation of significant improvement in the PPD values. A further explanation for the significantly less PPD in the AM group in contrast to the PRF may be that GTR creates a more resistant attachment than the graft [28].

Our investigation reported an overall rise in the relative attachment level (RAL) values from baseline to three months follow-up and from baseline to six months follow-up in both groups. This can be attributed to the coronal shift that occurs in the attachment apparatus with coronally anchored sutures, which hold the gingival margin in the new advanced position for 21 days, thereby eliminating any micromovement during the healing phase, and to the regenerative capacity of both the elements. The two groups did not significantly differ from one another in RAL over time, but the comparison within the groups between baseline and six months showed a statistically significant difference.

The increased RAL in Group II could be ascribed to the close resemblance between AM and the oral mucosal basement membrane. AM contains a variety of laminin types that can promote tissue adhesion, regeneration, angiogenesis, and tissue preservation, which aids in the healing of periodontal tissues, leading to a drop in clinical attachment level (CAL) and PPD [29].

The width of keratinized gingiva (WKG) increased from baseline to three months, and no significant change was seen in WKG from three months to six months of follow-up in both groups. Mean (SD) WKG increased from 1.52 (0.47) at baseline to 2.19 (0.36) at three months follow-up and remained stable at six months in Group I, whereas in Group II, the WKG increased from 1.63 (0.52) at baseline to 2.35 (0.55) at three months and remained the same at six months follow-up. These results are under the studies of Eren G et al. [25] and Tunali et al. [30], who had shown that PRF increased WKG the same as the gold standard subepithelial connective tissue graft (SCTG). Padma R et al. [16] reported that the addition of PRF to coronally advanced flap (CAF) augments the WKG in a split-mouth study design. This may be explained by the released growth factors from platelets trapped in a fibrin clot, which stimulate the development of gingival or periodontal fibroblasts.

When compared to CTG, Ghahroudi AA et al.'s [20] study reported an increase in the width of keratinized gingiva with AM. Along similar lines, our research also revealed an increase in WKG with AM. It is caused by the keratinocyte growth factor that AM releases, which may encourage epithelial cell keratinization and aid in the mucogingival junction's stability [20].

The strengths of the study include the use of the VISTA technique, which offers advantages such as minimal trauma to the gingiva, preservation of the interdental papilla's integrity, and improved aesthetic outcomes [13]. Additionally, PRF's biological properties, including the gradual release of cytokines and growth factors, contribute to enhanced wound healing [12]. AM's composition, which resembles the oral mucosal basement membrane, also supports its effectiveness in promoting tissue adhesion, regeneration, and angiogenesis [19].

Clinically, both amniotic membrane and platelet-rich fibrin used with the VISTA technique demonstrated predictable improvements in the treatment of Cairo’s RT1 gingival recession defects. As comparable outcomes were observed between groups, material selection may be guided by practical considerations. While platelet-rich fibrin provides the benefits of an autologous biomaterial, it requires venipuncture and chairside preparation. In contrast, the amniotic membrane offers advantages such as lower cost, ready availability, and elimination of patient anxiety associated with needle pricks during PRF preparation. Therefore, both approaches represent effective and minimally invasive options for achieving favorable root coverage outcomes in routine clinical practice.

Several limitations should be acknowledged when interpreting the outcomes of the present trial. Although 62 gingival recession sites were treated, these were contributed by only seven patients, resulting in a clustered study design with multiple sites nested within individuals. Such intra-patient clustering may introduce correlation among observations, thereby reducing statistical independence and effectively limiting the sample size at the patient level, which may influence the precision and generalizability of the findings. In addition, the study was conducted at a single center with a relatively small patient cohort and a short follow-up period, further restricting external validity. The analysis also did not account for gingival tissue thickness, a factor known to influence root coverage outcomes and periodontal wound healing.

Future investigations should therefore include larger, adequately powered multicenter randomized controlled trials with longer follow-up durations, incorporation of patient-level sample size calculations, and statistical approaches such as mixed-effects models to address clustered data. Furthermore, evaluation of the gingival phenotype and histological assessment of regenerated tissues may provide deeper insight into the biological mechanisms underlying clinical outcomes.

## Conclusions

The current study found that while both PRF and AM proved to be similarly efficient materials in terms of recession coverage, the amniotic membrane produced a better overall result and showed no negative responses.

PRF formation lengthens the surgical procedure overall, necessitates costly equipment that raises the patient's expense, poses a risk of blood-related complications that require extra care, and, above all, increases the patient's anxiety and fear of the needle prick used to withdraw blood before surgery. While amniotic's self-adherence shortens surgical time and makes it readily accessible at low cost, it is a viable alternative to PRF and other autografts for both patients and surgeons.
